# Impact Assessment of the Allergy Fact Checker, a Clinical Decision Support Tool for Noninvasive Beta-Lactam Antibiotic Allergy Label Delabeling: Protocol for a Multicenter Crossover Cluster-Controlled Study

**DOI:** 10.2196/86056

**Published:** 2026-03-27

**Authors:** Liesbeth Gilissen, Greet Van De Sijpe, Isabel Spriet, Rik Schrijvers

**Affiliations:** 1 Allergy and Clinical Immunology Research Group, Department of Microbiology, Immunology and Transplantation, KU Leuven, Herestraat 49, Leuven, Flanders, B-3000, Belgium, 32 16332211; 2 Department of Pharmaceutical and Pharmacological Sciences, Clinical Pharmacology and Pharmacotherapy, KU Leuven, Leuven, Belgium

**Keywords:** antibiotic allergy label, antimicrobial stewardship, beta-lactams, clinical decision support, delabeling, drug allergy, electronic patient records, noninvasive delabeling, medical records, penicillin

## Abstract

**Background:**

Beta-lactam allergy labels (BLALs), especially penicillin allergy labels, are frequently recorded in hospitalized patients and are associated with increased use of broad-spectrum and second-line antibiotics. Most BLALs are incorrect, but current allergy workups require invasive testing and specialized resources. We recently developed a strictly noninvasive, electronic patient record–embedded clinical decision support tool, the Allergy Fact Checker (AFC), which proactively identifies potentially incorrect BLALs by detecting uneventful re-exposures to the culprit or other beta-lactams since introduction of the BLAL.

**Objective:**

This study aims to evaluate the clinical, antimicrobial, and economic impact of the AFC in hospitalized adults with BLALs compared to the standard of care (no AFC).

**Methods:**

We are conducting a multicenter, open-label, crossover cluster-controlled study in 9 hospitals in Flanders, Belgium. All hospitalized adults with a BLAL are eligible, excluding patients in palliative care, discharged within 24 hours, or previously enrolled. Each hospital will alternate between intervention (use of the AFC) and control (standard practice) phases, separated by washout periods. The primary endpoint is cumulative guideline-concordant prescribing of first-line and/or narrow-spectrum beta-lactams following local antibiotic treatment guidelines, across predefined care windows up to day 100, expressed as a weighted per-patient proportion. Secondary endpoints include delabeling or refinement rate, beta-lactam tolerance, antibiotic switching, hospital length of stay, in-hospital and 3-month mortality, intensive care unit admission, readmission, multidrug-resistant organism colonization or infection, and costs. Based on prior data, we calculated that a total of 3285 participants are required to achieve 80% power to detect superiority of the intervention (4.6% vs 9.9% appropriate prescribing). Recruitment started in March 2025 and is ongoing.

**Results:**

The primary outcome will be analyzed using hierarchical mixed-effect models accounting for hospital-level clustering and period effects. Data collection will continue until 200 days after the last patient is discharged. This trial is expected to conclude in 2026. Ethical approval was obtained from the institutional review board in November 2024. Recruitment started in March 2025 and is ongoing. As of manuscript submission, more than 3000 participants have been enrolled across the participating hospitals. According to the prespecified protocol, an interim assessment of nuisance parameters will be performed in the following month to evaluate the initial design assumptions. Data collection is expected to continue until the end of 2026. The main study results are anticipated to be reported in 2027.

**Conclusions:**

This is the first multicenter European study evaluating a strictly noninvasive BLAL delabeling approach. If successful, this AFC tool could improve antimicrobial stewardship, reduce costs, and provide a scalable model for centralized allergy label management.

## Introduction

Drug allergy labels are an important tool to improve patient safety. They aim to reduce potential harmful re-exposure by alerting health care providers (HCP) of a prior allergic reaction. However, labeling is often insufficiently accurate, and an incorrect label may even cause harm through unnecessarily restraining patients from a first-choice treatment. Antibiotic allergy labels are the most frequently reported drug allergy labels [1]. Especially the prevalence of beta-lactams, which are first-choice treatments in most bacterial infections because of their spectrum, safety, low cost, and availability, is high. In US studies, the prevalence of beta-lactam allergy labels (BLALs) reported in electronic patient records (EPR) ranges between 9% and 16% [2-6], whereas European studies report a significantly lower prevalence of 1% to 5% [7-10]. In our center, covering over 1 million patients over an 8-year period, the observed BLAL prevalence was 2%, with rates varying from 1% in outpatient settings to 6% among hospitalized patients [11].

Most BLALs are incorrect. Upon a complete drug allergy workup, which includes a thorough clinical history, invasive skin and drug provocation testing, the majority of BLAL, especially penicillin, can be invalidated ranging from 95%‐99% of patients with a penicillin allergy label in United States and Australian studies [1-9], to 77%‐91% in European Union (EU) studies [10,11].

The impact of BLAL, irrespective of the correctness of the label, has been studied primarily in non-EU settings and mostly for penicillin. Penicillin allergy labels were associated with increased use of second-line and broad-spectrum antibiotics, a delay in time-to-first-dose upon admission [1,6,12], and increased odds for infection with antibiotic-resistant organisms [13-15]. Moreover, an increased length of hospital stay (LOS), mortality, intensive care unit (ICU) admission rate [5,16,17], and a higher economic cost [18-20] were found. Recent work from our group suggested that BLALs have limited impact on individual clinical outcomes (ie, no increased mortality, ICU admission rate, or increased LOS), although their effect on antibiotic use remains in line with findings from non-EU studies [21].

These concerns led to the emergence of invasive delabeling protocols for penicillin allergy labels in the United States and Australia [22,23]. Current approaches use risk stratification based on clinical history and direct challenges (ie, in-hospital, but without prior skin tests) with penicillins or alternative beta-lactams in identified low-risk patients [24,25]. The efforts demonstrate to be cost-effective [22] and impact antibiotic use, shifting more to first-line beta-lactams and narrow-spectrum penicillins [26]. However, a relevant fraction of readministrations was accompanied by a repeat hypersensitivity reaction (3.8%), of which a minority (<1%) were severe [26]. This illustrates the delicate balance between indirect harm through incorrect BLAL and repeat iatrogenicity through invasive delabeling protocols themselves.

An often forgotten step in the delabeling process that encompasses the lowest risk for repeat iatrogeny is noninvasive delabeling. Here, without skin or drug provocation testing, but solely by using patient questionnaires and information from tolerated antibiotic exposures, discordances in the EPR are exploited to the advantage of the patient [9,27-29]. Our group prospectively evaluated in an EU context a noninvasive delabeling protocol in adult internal medicine inpatients with a BLAL [30], combining (1) an allergy-oriented clinical history, (2) an EPR search to identify incorrect BLAL and/or uneventful re-exposures to beta-lactams, and (3) a systematic primary care physician and community pharmacist contact. Using this approach, 38% of BLALs could be delabeled, and another 27% could be refined, largely driven by EPR findings. In the next step, we optimized the EPR search by developing a semiautomated clinical decision support (CDS) tool, designed to identify those patients with documented re-exposure without allergic reaction. A proof-of-concept study, limited to penicillin allergy labels, demonstrated the feasibility and efficacy of this novel CDS tool, identifying nearly 10% of alleged penicillin-allergic patients and showing approximately sevenfold higher odds of delabeling after its introduction [31]. Importantly, the impact on prescription behavior and major clinical outcome parameters is unknown and would be the basis for broad roll-out. Therefore, we propose to perform the first multicenter study on noninvasive BLAL delabeling, using an EPR-embedded, semiautomatic, intelligent “Allergy Fact Checker (AFC)” CDS tool.

## Methods

### Aim

This study aims to evaluate the clinical and economic benefits of a noninvasive CDS tool, critically assessing BLALs the AFC, while following local antibiotic treatment guidelines. Specifically, we will assess its impact on antibiotic use, as well as clinical, antimicrobial, and economic end points in hospitalized adults with BLAL. We hypothesize that a relevant fraction of BLAL can be rationalized by the AFC, thereby improving antibiotic use and costs without impacting morbidity or mortality.

### Design

A multicenter, open-label, crossover cluster-controlled trial will be conducted to compare the use of our AFC on BLAL (intervention, I) and subsequent antibiotic prescription with current practice (control, C, ie, no AFC). All hospitalized adults with one or more BLAL will be included, except those previously enrolled in this study, discharged within 24 hours, or in palliative care. The primary endpoint is cumulative guideline-concordant prescribing of first-line and/or narrow-spectrum beta-lactams (based on the IGGI [Infectiologiegids/Guide d’Infectiologie] guidelines of the Belgian Society for Infectiology and Clinical Microbiology) across predefined care windows up to day 100, expressed as a weighted per-patient proportion. Within each predefined assessment window, prescribing is scored in an any or ever binary manner, and a cumulative weighted score per patient is calculated across applicable windows. Secondary endpoints include proportion of delabeled and refined BLALs; beta-lactam antibiotic tolerance; need to switch antibiotics; in-hospital and 3-month posthospitalization mortality; LOS; 3-month readmission rate; ICU admission rate; colonization and/or infection with methicillin-resistant *Staphylococcus aureus*, vancomycin-resistant *Enterococcus*, *Clostridium difficile* (*C. diff*); antibiotic and hospitalization cost, time, and resources needed for the AFC; and physician appraisal of the approach.

We chose a mono-faceted intervention without an awareness campaign or educational component (eg, e-learning for physicians on BLAL labeling and delabeling) to evaluate the impact of the AFC itself, enhancing its extrapolation afterward. To correct for interhospital variability, we choose a clustered crossover design. The crossover design also accounts for seasonal variability, for example, in overall antibiotic use.

Allocation is performed on the hospital level (participating sites) before the start of this study to prevent contamination of effect between the intervention and control phases (ie, so that physicians will not receive information through the AFC while having to use conventional means for different patients at the same time). The allocation is nonrandomized and operationally predefined based on the availability of a single trained research team responsible for on-site implementation across hospitals. Inclusion periods are sufficiently long and distributed over calendar time to account for seasonal variation. A crossover cluster-controlled design was used to reduce potential confounding related to temporal and organizational factors in clinical practice, such as variation in staffing and workload among treating physicians. Residual temporal and cluster-level confounding will be further addressed in the statistical analysis by including study period and hospital as model terms.

**Figure 1.**
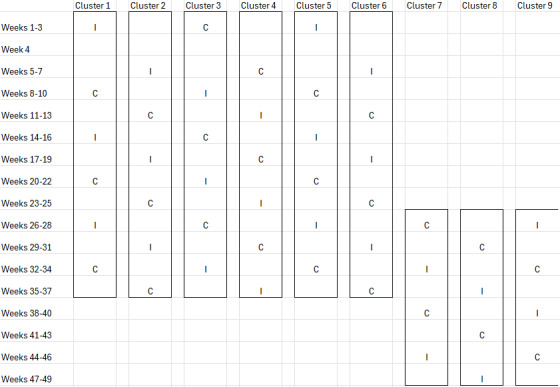
Schematic representation of this study’s design.

During separate intervention phases, hospital physicians will receive the intervention, that is, BLAL CDS. Each of the crossover periods will be 3 weeks long. However, a washout period of 3 weeks is also taken into account to allow the patient population to be sufficiently “new” before the cluster transitions to the next condition. We will use the sequences I-x-C-x, x-I-x-C, C-x-I-x, and x-C-x-I (where I=intervention, ie, use of the AFC, and C=control, ie, standard of care [SOC], no use of the AFC, and x=washout period), and each cluster will be allocated to one of these sequences (Figure 1). Information about the assigned sequence is available to all people and groups involved in the research (ie, unblinded, open study).

### Setting

Patients will be recruited in 9 participating centers across Flanders, that is, the University Hospitals of Leuven (UZ [Universitair Ziekenhuis] Leuven), and the general hospitals of Pelt (Noorderhart Mariaziekenhuis), Mol (Heilig Hartziekenhuis), Herentals (AZ [Algemeen Ziekenhuis (general hospital)] Herentals), Turnhout (AZ Turnhout), Izegem (Sint-Jozefskliniek), Halle (AZ Sint-Maria Halle), Tienen (RZ Heilig Hart Tienen), and Malle (AZ Voorkempen). The expected recruitment period, from First Patient In to Last Patient In, is 11 months, starting March 10, 2025. The total inclusion period from First Patient In to Last Patient Out is expected to take approximately 18 months. The total period of intervention plus follow-up for every single patient depends on the LOS, that is, until 200 days after discharge from the hospital, to collect the secondary end point parameters: in-hospital mortality, 3-month posthospitalization mortality, LOS, and 3-month readmission rate. Patients who are still in the hospital when the end of the recruitment period is reached (estimated February 2026, when the needed sample size was reached) will be excluded. For comparison, in a recent retrospective study in UZ Leuven, involving 21,999 patients with pneumonia, pyelonephritis, infections associated with heart, kidney, liver, or lung transplantation, appendectomy, coronary artery bypass grafting, and total knee or hip replacement, the average LOS in UZ Leuven for patients with BLAL was 21 days (SD 39) [21].

### Study Procedures

**Figure 2.**
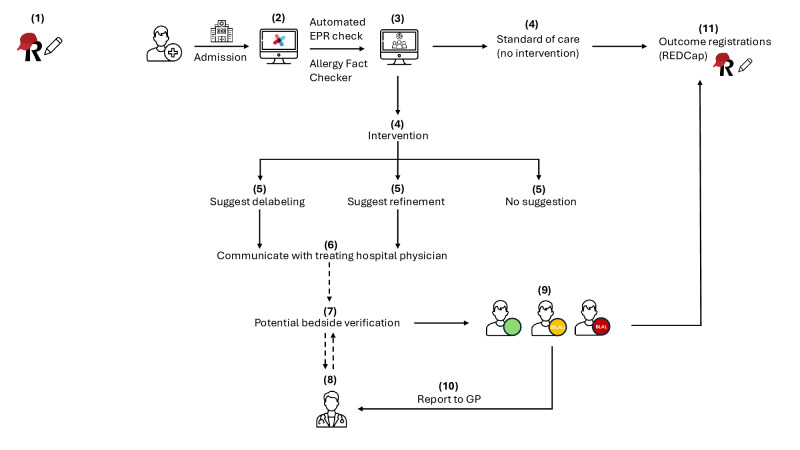
Schematic representation of the intervention vs standard of care. (1) Report baseline measurements eCRF; (2) automated search in the hospital EPR for re-exposure; (3) study team members receive AFC results on working list; (4) inclusion of patient in either standard of care or intervention (depending on scheme in Figure 1); (5) EPR check to identify BLAL for delabeling, refinement, or no action; (6) action is communicated to treating hospital physician (structured notes); (7) and (8) treating hospital physician can perform bedside verification and/or contact primary care HCP; (9) formal report of the results in the EPR; (10) letter to the primary care HCP; and (11) outcome parameters are followed up and scored in eCRF. AFC: Allergy Fact Checker; BLAL: beta-lactam allergy label; eCRF: electronic case report form; EPR: electronic patient record; GP: general practitioner; HCP: health care provider; REDCap: Research Electronic Data Capture; Vanderbilt University.

Study procedures and their timing are summarized in this study’s flowchart (Table S1 in Multimedia Appendix 1). Figure 2 shows a schematic representation of the different steps of the intervention vs SOC. All study-specific procedures will be carried out by the team of the coordinating investigator at the different participating sites to minimize time consumption from principal investigators. This allergy-trained central study team consists of an allergy specialist (RS), an internal medicine specialist in training for allergy and clinical immunology and dedicated to this study (Dries Wets, MD), a biomedical scientist acting as a data manager (Nghia Nguyen, MSc), and a pharmacist-PhD trained in allergy (LG). This study’s team (LG, RS, Dries Wets and Nghia Nguyen) evaluates the cases that are flagged and ensures communication with the treating physician (Figure S1 in Multimedia Appendix 1).

### Patient Inclusion

Potential participants will be identified and recruited during hospitalizations in one of the participating sites. An EPR-embedded clinical rule identifies in near-real time all hospitalized patients with a BLAL in the allergy module. After checking inclusion and exclusion criteria, all eligible patients will be included in this study.

### Ethical Considerations

This clinical study was initiated after evaluation and approval by the Research Ethics Committee UZ/KU Leuven (S68439, protocol version 5, July 2, 2025), and after consultation with the ethics committees (EC) of each participating center in Belgium (Commissie voor Medische Ethiek [CME] Noorderhart [S504409]; EC St-Jozefskliniek Izegem [S504492]; CME Heilig Hart Ziekenhuis Mol [S504443]; CME AZ Herentals [S504441]; CME AZ Turnhout [S504448]; CME AZ Sint-Maria, CME RZ Tienen and CME AZ Voorkempen [approval granted; S-numbers pending]).

This study was classified as a noninterventional quality improvement project. The project does not introduce a new clinical treatment or experimental procedure but consists of systematically identifying discordances in existing electronic health records (ie, documented BLALs despite proven prior tolerance) and communicating this information to the treating physician through a decision-support process. The final clinical decision always remains with the treating physician and follows existing international and national guidelines. No additional diagnostic or monitoring procedures are imposed, and no automatic delabeling is performed by this study’s team. Therefore, this study does not mandate changes in patient management but supports standard-of-care decision-making. Under Article 2, §8 of the Belgian Law of 7 May 2004 [31], this project qualifies as a noninterventional study, as medications are prescribed according to routine clinical practice, no additional diagnostic or monitoring procedures are imposed, and epidemiological methods are used for analysis. Moreover, the risks for participants are minimal and proportionate to the anticipated benefits. However, for methodological clarity, the term “intervention” is used in this protocol but refers solely to an informational decision-support process and not to a patient-level clinical intervention.

Therefore, individual informed consent is not required. Instead, a structured patient notification and opt-out procedure is implemented in accordance with institutional policies[32].

To protect participant privacy, all study data are recorded in a secure electronic data capture system (REDCap [Research Electronic Data Capture]). Only authorized study personnel have access to identifiable data. For analysis, data are pseudonymized and stored on secure institutional servers in accordance with applicable data protection regulations, including the General Data Protection Regulation.

Participants do not receive any financial compensation or incentives for participation in this study.

### Informed Consent

Individual informed consent is not required, as this study was classified as a noninterventional quality improvement project.

Moreover, obtaining individual informed consent was considered inappropriate for several reasons: it could introduce bias by alerting physicians and/or patients of their label, potentially leading to behavioral changes compromising study validity, particularly in the control group; this study poses minimal risk, with potential disadvantages proportional to the expected benefits; the intervention targets physician prescribing behavior, not individual patients; a complete dataset is essential, excluding nonconsenting patients would limit validity and representativeness.

Instead of traditional informed consent, a structured patient notification and opt-out procedure is applied. Participants are informed through a study information letter made available via the EPR user application (Mynexuzhealth) and via the publicly accessible study webpage [33]. This study is also publicly announced through posters displayed in participating hospitals (Figure S2 in Multimedia Appendix 1). Patients are explicitly informed that they can withdraw at any time without consequences for their treatment by contacting this study’s team, as indicated in the information letter. If the dataset is not yet locked, previously collected data will be removed, and no further data will be collected. Any proposed label adjustments are actively communicated to both patients and their HCPs through the EPR (cf. Infra, communication of the AFC results, intervention group, if applicable).

These procedures were reviewed in depth and formally approved by the central ethics committee as well as by the local ethics committees of all participating sites.

### Inclusion Criteria

The inclusion criteria are hospitalized patients and BLAL (for one or more beta-lactams).

### Exclusion Criteria

The exclusion criteria are being aged younger than 18 years, previous enrollment in this study, patients receiving palliative care, and patients expected to be discharged within 24 hours.

### Baseline Measurements

For each patient, baseline data will be recorded in the electronic case report form using the REDCap (cf. Infra). The collected information will encompass sociodemographic details, clinical data, and specific information on the BLALs as registered in the EPR. Sociodemographic information will include age, gender, and contact details of both the general physician and the community pharmacist. Clinical information will capture the reason for admission and, if applicable, the condition requiring antibiotic treatment, categorized according to the *ICD* (*International Classification of Diseases*) codes. Additionally, any antibiotics (Anatomical Therapeutic Chemical codes J) already administered during the current hospitalization will be documented. To assess comorbidity burden, the presence of specific conditions contributing to the Charlson Comorbidity Index will be recorded, including myocardial infarction, congestive heart failure, peripheral vascular disease, cerebrovascular disease, dementia, chronic pulmonary disease, rheumatologic disease, peptic ulcer disease, liver disease, diabetes, hemiplegia or paraplegia, renal disease, malignancy (localized or metastatic), leukemia, lymphoma, and acquired immunodeficiency syndrome. Information on BLALs will be extracted from the allergy registration module within the EPR. This will include the registration date of the BLAL, the specific drug involved (Anatomical Therapeutic Chemical code), the onset date of the allergic reaction, reported symptoms, and the source of information. Additionally, the probability of label accuracy, as indicated by the reporter, will be documented.

### AFC CDS Tool (Intervention Group)

A clinical rule embedded in the EPR checks in near-time for re-exposure to the culprit antibiotic or class member, from the date of BLAL registration to the date of patient inclusion (the AFC). The AFC is incorporated into the hospital information system as an “if-then” algorithm, using real-time structured data available in the EPR. The AFC incorporates information from the electronic prescribing module in the EPR and uses an integrated natural language processing tool to identify re-exposures since the introduction of the BLAL in the various EPR records. Additionally, it lists data on coadministration of antihistamines, glucocorticoids, or epinephrine during the re-exposure period, up to 24 hours after the last antibiotic dose. These medications are considered proxies for possible allergic reactions and help inform the subsequent review process. The results of the screening, that is, the clinical rule alerts, are compiled on a structured worklist in the hospital information system for secondary review by a specified trained person, that is, a member of the study team in the current study, before alerting the treating physician [24]. Validated flowcharts are used to support the review, ensuring a standardized and systematic approach. Schematic algorithms are used to guide the actual delabeling or refinement advice based on the allergy label, subsequent exposure, and consequences linked to this exposure (Figure S3 in Multimedia Appendix 1).

Consulting primary care data could not yet be automated due to the lack of interoperability between different EPRs. In order to retain a focus on the impact of the AFC alone, we did not systematically obtain information from the primary care HCP (physician and/or pharmacist) on beta-lactam re-exposure. In our pilot study [30], this step provided only a minor contribution.

Based on the collected information, BLALs are allocated to 3 groups: to delabel, to refine, or to leave as such. An incorrect BLAL is assigned when patients reported a clearly nonallergic adverse drug reaction, when a negative rechallenge or uneventful re-exposure was identified since registration of the BLAL; a correct BLAL is assigned when an allergic reaction was observed in the hospital or when positive skin and/or provocation testing was found; and an undetermined BLAL is assigned when there is no proof of correctness or incorrectness of the BLAL. Allocation is performed by a member of this study’s team, and if needed, discussed and validated with the principal investigator.

### BLAL Adjustment (Intervention Group, if Applicable)

In the case of a correct or undetermined BLAL, no action will be taken. In case of an incorrect BLAL, a suggestion will be sent to the treating physicians in the EPR (“follow-up note”), to delabel (re-exposure to the culprit found) or to refine (harmless exposure to nonculprit beta-lactams, excluding monobactams or carbapenems found) the BLAL accordingly. The note will include a summary of the algorithm’s findings, a suggestion for review of the label in consultation with the patient, a reference to the in-house delabeling procedure (written and video), and contact details for questions and additional information. If the BLAL is not changed in the following 24‐72 hours, the treating physician will be contacted by phone to discuss potential delabeling; reasons to discard the advice will be noted in the electronic case report form (as part of the process evaluation) and in the formal study report (cf. Infra). A distinction is made between definite tolerated re-exposure and presumed tolerance. The former is scored in case of documented administration through the electronic prescribing module (in which prescription and registered administration are required), in the EPR, and/or when bedside uneventful re-exposure could be orally confirmed by the treating hospital physician. The latter is scored when free-text notes or ambulatory prescriptions of antibiotic delivery are identified in the EPR, in the absence of EPR-registered bedside verification by the treating hospital physician.

### Communication of the AFC Results (Intervention Group, if Applicable)

In case of an incorrect BLAL, this study’s member will make a formal report of the AFC result in the EPR (Figure S4 in Multimedia Appendix 1), available for hospital physicians and, after validation of the coordinating investigator, also for the patient in the Mynexuzhealth app. This report is also sent to the primary care HCP via e-letter through the eHealth platform. The report will include a summary of our findings and, if applicable, label adjustment. Patients are referred to their primary care HCP, this study’s information letter for participants, and this study’s webpage with contact details of this study’s team for further questions and/or disagreement with the label adjustment.

### Follow-Up or Outcome Measurement

To evaluate the effectiveness and safety of the intervention, information on antibiotic prescriptions, BLAL details, safety indicators, clinical outcomes, microbiological end points, and economic factors will be extracted from the EPR at days 30, 100, and 200. Antibiotic prescription will be monitored throughout the follow-up period. BLAL details will include the outcome of the assessment, along with any newly recorded or relabeled BLALs. The primary outcome is assessed per patient at the predefined time points. Inconclusive outcomes will be single-blinded evaluated by 2 independent infectious disease specialists who, upon disagreement, discuss the outcome with a third senior infectious disease specialist for a consensus. In the absence of doubt regarding the use (or not) of a first-line regimen, the scores are scored (unblinded) by the central study team (n=2). Safety parameters will be evaluated based on antibiotic tolerance and the need to switch antibiotics during treatment. Clinical outcomes will be assessed by measuring the number of days until in-hospital mortality, mortality at 3 months posthospitalization, LOS, and ICU admission rate. Microbiological end points will include colonization and/or infection of methicillin-resistant *Staphylococcus aureus*, vancomycin-resistant *Enterococcus*, and *C. diff.* economic end points, focusing on the societal perspective, will capture the cost of antibiotic treatment and hospitalization, as well as the time and human resources required. However, detailed economic analysis will be the subject of a separate protocol. No patients will be lost to follow-up within participating hospitals, as this study relies exclusively on back-office EPR data extraction. Outcomes are captured through the shared EPR platform “Clinical Workstation” (CWS, Nexuzhealth), a multihospital EPR network used by several tertiary and regional Belgian hospitals. However, antibiotic exposure and readmissions are only captured for hospitals using this EPR network; outpatient antibiotic use and care in nonnetwork hospitals are not captured. Moreover, survival is centrally registered and automatically linked to the patient’s Belgian social security number, allowing us to reliably follow up on this parameter irrespective of subsequent hospitalizations.

### Missing Data

Baseline variables used by the AFC tool are routinely collected in the electronic health record and are therefore expected to be minimally missing. For follow-up outcomes, mortality is centrally collected (and therefore not expected to yield missing data). However, intercenter variability in data capture may occur. Therefore, for time-to-event outcomes (survival), patients will be censored at the last known follow-up in accordance with standard survival analysis methods. For binary and continuous secondary outcomes (eg, antimicrobial resistance and antibiotic use), we will assume data are missing at random and apply multilevel multiple imputation, using Rubin rules. For the window-based prescribing outcomes, windows without in-hospital systemic antibiotic exposure are considered not applicable (“no opportunity”) rather than missing and are excluded from the denominator of the cumulative primary end point. Patients without any applicable windows (no in-hospital antibiotic exposure in any assessment window up to day 100) will be excluded from the primary end point analysis, as the cumulative guideline-concordance proportion is not defined for these patients. The number and proportion of such patients will be reported by study phase. The proportion and patterns of missing data will be reported accordingly, and sensitivity analyses will be conducted using complete-case analyses and worst-case assumptions to assess the robustness of findings.

### Sample Size Determination

The main aim of our study is to establish the assumed superiority of using the AFC over SOC in increasing the proportion of patients with first-line antibiotics prescribed. Therefore, we look at the proportion of patients receiving first-line or narrow-spectrum beta-lactam antibiotics within the total number of patients receiving antibiotics. The crossover design accounts for seasonal as well as intra- and interhospital differences in overall antibiotic use. Nevertheless, overall antibiotic use will be monitored at the participating sites during this trial’s period.

The following assumptions were applied.

For the control arm, in a recent retrospective study in UZ Leuven, involving 21,999 patients with pneumonia, pyelonephritis, infections associated with heart, kidney, liver, or lung transplantation; appendectomy; coronary artery bypass grafting; and total knee or hip replacement, we investigated the difference in antibiotic use between patients with and without BLAL [21]. It showed that 10.5% of patients with a BLAL received amoxicillin despite their label. Based on the latter, we estimate that 10% of the patients in the control group already receive first-line antibiotics. We add a 5% increase due to indirect learning of our protocol because we cannot exclude that treating physicians learn to use discordances in the medical file for delabeling or refinement of the allergy labels, independent of our study and AFC tool. Considering around 30% of hospitalized patients receive antibiotics during their stay [34], this brings the percentage down to 4.6% in the control group.

For the intervention arm, we previously showed that our CDS approach has the potential to delabel 38% of BLALs [30]. We add a margin of 5% for physicians or patients who refuse to delabel or be delabeled [9]. Considering around 30% of hospitalized patients will receive antibiotics during their stay, this results in an overall estimate for the intervention group of 9.9%.

For the intra- and intercluster correlation (ICC), literature indicates a moderate ICC of 0.059 for antibiotic prescription behavior [35,36]. This estimate seems appropriate as moderate differences in antibiotic prescription behavior are expected within and between different physicians at participating sites, which mostly follow the IGGI guidelines, however, with some internal adjustments. Indeed, the most recent point prevalence survey of antimicrobial use and health care–associated infections in Belgian acute care hospitals (results of the global point prevalence survey and European Center for Disease Prevention and Control point prevalence survey) reports 77% compliance with IGGI guidelines [37]. Additionally, 3 participating sites are part of the same Hospital Outbreak Support Team network, that is, Heilig Hartziekenhuis Mol, AZ Turnhout, and AZ Herentals in the Hospital Outbreak Support Team Network Kempen, which has the specific goal to align antimicrobial stewardship. All centers use the same EPR (CWS). The major difference would be expected between UZ Leuven, as an academic hospital, and the other participating sites, as regional hospitals. The AFC is currently only available in the CWS EPR system, and therefore, we were unable to include other academic centers as well. A sensitivity analysis of academic vs regional hospitals will be performed.

Considering the number of hospitalizations per year in each center and a prevalence of 6% of BLALs [8], we expect an inclusion rate of 3285 participants.

Sample size and power calculations were carried out according to the methods described by Hemming et al [38], using the Shiny CRT Calculator [39]. Based on the design given in Figure 1, assuming a discrete time decay (allowing correlations between periods to decay with each period), a within-period ICC of 0.059 and an autocorrelation (ratio of between-period and within-period ICC) of 0.6, this study will have 80% power to detect a statistically significant difference between control and intervention using a 2-sided significance level of 5%, assuming 4.6% and 9.9% of patients to have the primary end point in the control and intervention group, respectively. This sample size calculation is based on patient-level appropriate prescribing proportions and is considered a conservative approximation for the cumulative window-based primary end point.

In case key assumptions differ importantly from those used in the initial calculations, we will implement a preplanned sensitivity and adaptation strategy focused on nuisance parameters rather than treatment effect estimates. Once the initial target number of inclusions has been reached, we will conduct a blinded (or arm-masked) internal review of (1) overall primary end point incidence, (2) observed within-period ICC and between-period correlation structure, and (3) completeness of follow-up and outcome ascertainment across centers and periods. In parallel, we will run sensitivity power analyses across plausible ranges of ICC or autocorrelation to quantify the robustness of power under departures from assumptions. If this review indicates that statistical power is likely to be compromised (eg, lower-than-expected event rate or higher-than-expected clustering), we will adapt by extending recruitment and/or adding additional crossover periods and/or centers, while keeping the intervention allocation scheme and primary outcome definition unchanged, depending on the available human resources to perform this. Any such modifications will be documented as protocol amendments and reflected in an updated statistical analysis plan. Importantly, to avoid operational recruitment gaps and preserve feasibility, recruitment will continue while this internal review is performed, and the adaptation decision will be based on updated nuisance-parameter estimates rather than interim efficacy signals.

### Statistical Analysis

The primary endpoint is cumulative guideline-concordant use of first-line and/or narrow-spectrum beta-lactam antibiotics according to IGGI-based local guidelines, assessed across predefined assessment windows up to day 100 (days 1, 30, and 100). Within each window, guideline concordance is scored at the patient level in an any or ever manner as 1 if at least one in-hospital systemic antibiotic course is guideline-concordant and 0 if otherwise. If multiple courses occur within a window, the window is considered concordant if any course meets guideline criteria. This any or ever approach was chosen in line with the quality-improvement objective of the AFC intervention, capturing whether appropriate first-line therapy was achieved within a care window while limiting instability due to multiple within-episode treatment changes. Windows without in-hospital systemic antibiotic administration are considered not applicable (“no opportunity”) and are excluded from the denominator.

For each patient, a cumulative weighted guideline-concordance score is calculated at day 100 as the proportion of applicable windows with guideline-concordant therapy (*sᵢ/nᵢ*). The primary comparison between intervention (AFC) and control will use a binomial generalized linear mixed-effects model with logit link, modeling *sᵢ* out of *nᵢ*, including fixed effects for study phase and period, and a random intercept for hospital to account for clustering in the crossover design. A prespecified adjusted model will additionally include age, sex, and the Charlson comorbidity index. Patients without any applicable windows will be excluded from this analysis and reported separately. The primary estimand targets the period during which the AFC is expected to plausibly influence prescribing through contemporaneous decision support and short-term label adjustment.

Secondary endpoints include guideline-concordant beta-lactam use at each individual assessment window (including day 200), delabeling or refinement rate, beta-lactam tolerance, antibiotic switching, ICU admission, readmission, resistant organism outcomes, LOS, mortality, and cost outcomes. Outcomes at day 200 are analyzed as secondary durability measures, as longer-term prescribing may be influenced by label reappearance or documentation changes unrelated to the index intervention. Secondary endpoints will be analyzed using hierarchical mixed-effects models appropriate to outcome type (binary, continuous, or time-to-event), with fixed effects for study phase and period and clustering by hospital. Process evaluation outcomes will be analyzed descriptively.

Predefined subgroup analyses will assess potential effect modification by including phase-by-subgroup interaction terms in the primary model. Subgroups of interest are BLAL type (penicillin vs other labels), reported reaction severity category, time since label registration, infection type (prophylaxis vs therapeutic indication), ICU admission status, and hospital type (academic vs regional). Subgroup analyses will focus on the primary endpoint and are considered exploratory and hypothesis-generating. 

Moreover, because the AFC is expected to influence prescribing primarily through label delabeling or refinement, we will perform an exploratory implementation analysis within the AFC phase. Patients will be classified as with implementation if the BLAL was delabeled or refined after the AFC recommendation, and as not with implementation if otherwise. The cumulative primary endpoint (*sᵢ/nᵢ* up to day 100) will be summarized by implementation status; any between-group comparisons will be considered descriptive and interpreted cautiously, as implementation is a postallocation process measure and may be confounded.

No formal adjustment for multiplicity will be applied. Secondary and subgroup analyses will be interpreted with caution.

Sensitivity analyses will include evaluation of alternative covariance structures for within-cluster and within-period correlation (eg, exchangeable or autoregressive), with model selection guided by goodness-of-fit criteria, and estimation using generalized estimating equations with robust SEs to obtain marginal effect estimates and assess robustness to modeling assumptions. As a robustness check, we will report the proportion of patients with any recorded in-hospital follow-up within the shared EPR network (CWS) by study phase and period.

A detailed statistical analysis plan and data management plan will be finalized before database lock.

### Quality Assurance

In order to ensure the same quality and safety standards in patient care for clinical research as commonly applied by the research team in its regular activities, the research team shall comply with the following obligations: (1) the research team will use trained and qualified employees or contractors to manage and coordinate this study; (2) the sponsor will ensure that this (multicenter) study’s reporting is reliable and valid, statistically accurate, ethical, and unbiased; (3) the research team will not grant incentives, other than standard compensations and reimbursement of costs, to this study’s participants or to participating site’s staff that would compromise the integrity of the research; (4) the research team is responsible for monitoring and evaluating the quality, safety, and ethics of this study and will respect the participating site’s policies and processes when performing such monitoring and evaluation activities; and (5) the research team will protect the privacy and confidentiality of this study participants in accordance with all applicable laws.

A dedicated postdoctoral researcher and pharmacist (LG) will be in charge of overall trial (quality) management, and monthly coordinating investigator study team meetings will be organized.

Moreover, a dedicated advisory board is installed for this project, consisting of all principal investigators, other experts, and additional stakeholders for further valorization of our approach. This advisory board will be informed and consulted about the project progress at least every 6 months (ie, 6 times in total). A dedicated Teams (Microsoft Corp) environment will be created to ensure efficient communication during and in between the semestral board meetings. The overall role of the advisory board is to ensure the project goals, milestones, and deliverables are reached in time, to ensure the technicalities of the tool match the needs and criteria for the respective stakeholder group, and to promote usage of the tool postproject in the relevant stakeholder groups.

## Results

Recruitment for this multicenter crossover cluster-controlled study is ongoing across hospitals in Flanders, Belgium. Recruitment started in March 2025. As of March 2026, more than 3000 participants have been enrolled across 6 participating hospitals, which approximates the initially estimated sample size required for the planned power calculation.

According to the prespecified protocol, an interim assessment of nuisance parameters will be conducted in the following month to evaluate the initial design assumptions. Following this assessment, recruitment will continue with the participation of 3 additional hospitals, with data collection expected to continue until late 2026.

The final study database is anticipated to be locked in late 2026, after completion of follow-up. The prespecified statistical analyses will then be performed. The main study results are expected to be reported in 2027.

## Discussion

Noninvasive delabeling is an often-forgotten step in the process of correct BLAL labeling and delabeling. Previous work, including ours, has shown its potential. However, its impact on clinical end points is unclear. Here, a novel CDS tool, the AFC, embedded in the EPR and driven by an expert-elaborated algorithm, is expected to be a key tool for (proactive) noninvasive antibiotic allergy delabeling or refinement. This study aims to evaluate the clinical, antimicrobial, and economic impact of implementing the AFC for hospitalized adults with BLALs compared to SOC (no AFC). The AFC holds the potential to also optimize other (nonantibiotic, eg, iodinated contrast media and others) allergy labels. Moreover, it would offer additional arguments to support health data sharing and the development of an overarching patient record system, which would stimulate interdisciplinary communication. Additional valorization steps, such as leveraging our tool to nonhospital EPRs, would be relevant as improving BLAL evidently transcends the boundaries of primary, secondary, and tertiary care facilities. The heterogeneous primary care EPR antibiotic allergy labels are not yet linked to the hospital EPR in our country. Nevertheless, we believe the future integration and sharing of extramural medical data holds great promise and should remain a next step. A centralized registration module (in eHealth Belgium or through a European Allergy Module in the European Health Data Space) would provide a solution, but requires further optimization. However, additional elements need to be taken into account. First, the primary endpoint used a cumulative, window-based guideline-concordance measure with any or ever scoring up to day 100. This pragmatic definition aligns with the quality-improvement objective of the AFC intervention and captures whether appropriate first-line therapy was achieved within each care window, but it simplifies complex treatment courses and may overestimate concordance when multiple regimens occur within a window. Second, follow-up antibiotic use and readmissions are ascertained from in-hospital records across hospitals using the shared EPR platform (CWS), which includes several large Belgian hospitals. Care delivered outside this network and outpatient antibiotic use are not captured, which may lead to underestimation of total exposure and guideline concordance. If nondifferential between study phases, this would likely bias effect estimates toward the null; the degree of in-network follow-up capture will therefore be reported. Third, exposure to the AFC may directly or indirectly induce a learning effect, potentially reducing the observed difference between the intervention and control groups. To avoid overestimation of the AFC effect, we conservatively corrected for an estimated 5% increase in non-AFC–induced delabeling in the control group. Importantly, a learning effect is likely to occur in both groups over time; therefore, this correction should be regarded as an approximation allowing a more accurate estimation of the true AFC-specific impact. Residual confounding due to non-randomized allocation and temporal variation in clinical context cannot be fully excluded, although this risk was mitigated through the crossover cluster design and adjustment for period and hospital effects in the statistical analysis. In addition, truly allergic patients may lack documented allergy labels, and information on drugs given outside of the registered hospital context may be incorrect. Finally, the scalability of our AFC tool will depend on its ability to be integrated into other EPR software platforms. The AFC tool is based on the principle of discordant patient information. Exploiting these discrepancies to the benefit of the patients would allow for improved EPRs and is in line with the goal of a unified EPR.

## Supplementary material

10.2196/86056 Multimedia Appendix 1 Supporting figures and table.

## Abbreviations

AFC: Allergy Fact Checker
AZ: Algemeen Ziekenhuis (general hospital)
BLAL: beta-lactam allergy label
CDS: clinical decision support
CME: Commissie voor Medische Ethiek
CWS: Clinical Workstation
EPR: electronic patient record
EU: European Union
HCP: health care provider
ICC: intra- and intercluster correlation
*ICD*: *International Classification of Diseases*
ICU: intensive care unit
IGGI: Infectiologiegids/Guide d’Infectiologie of the Belgian Society for Infectiology and Clinical Microbiology
LOS: length of hospital stay
REDCap: Research Electronic Data Capture
SOC: standard of care
UZ: Universitair Ziekenhuis (university hospital)
