# Supplementary material for: Impact Assessment of the Allergy Fact Checker, a Clinical Decision Support Tool for Noninvasive Beta-Lactam Antibiotic Allergy Label Delabeling: Protocol for a Multicenter Crossover Cluster-Controlled Study
Source: JMIR Res Protoc. 2026 Mar 27;15:e86056. doi: 10.2196/86056 (PMC13026425; doi:10.2196/86056)
Supplement: Multimedia Appendix 1 — Supporting figures and table. [file resprot-v15-e86056-s001.docx]

**Figure S1.** Overview of actions performed by the study team.

**1/ Check attendance list** of hospitalized patients with an antibiotic allergy label using a speciality board (Specialty board < Allergy fact checker bundle < Rule ‘ALL allergielabel’)

**2/ Verify inclusion and exclusion criteria**: *older than 18 years old at time of admission; admitted to hospital for at least 24 hours; not included in study before; does not receive palliative care at time of admission; no opt-out for secondary use of medical data.*

**3/** If conditions are met, **include** patient in the study.

**SCENARIO: INTERVENTION GROUP**


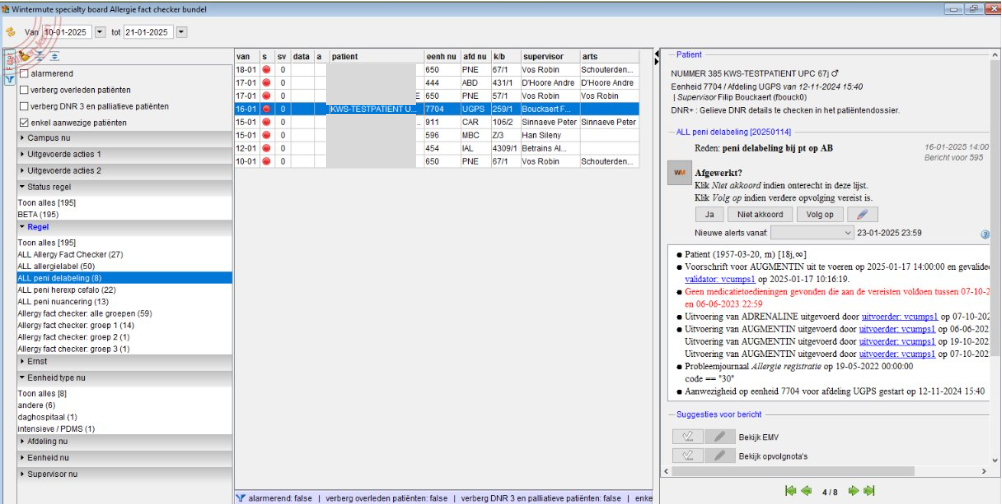


**4/ Check output** **of wintermute message**: date of allergy label; re-exposition (which drugs and when), possible administration of antihistamines/corticosteroids/adrenalin during re-exposure.

**5/ Check whether the index reaction occurred after administration of a penicillin other than benzylpenicillin, amoxicillin or amoxicillin-clavulanate.**

> *If yes*, dismiss the case and finish alert.

> *If no*, check the electronical medical prescription at the time of re-exposure to identify

red flags that indicate a possible allergic reaction, such as the administration of Solu-Medrol or Solu-cortef, antihistamines or adrenalin.

**6/ Review the follow-up notes and the notes in the care section to ensure that the re-exposure was well tolerated**: was an allergic reaction reported during the re-exposure?

> *If yes*, dismiss the case and finish alert.

> *If no*, create follow-up note in the medical record of the patient to suggest delabelling or to adjust the allergy label by the physician, if deemed appropriate by the physician.

**7/ Follow-up with physician**: if a delabeling/refinement decision has not been incorporated within 24 hours, call the treating physician for follow up.

**Figure S2.** Patient notification material (Dutch, original version).


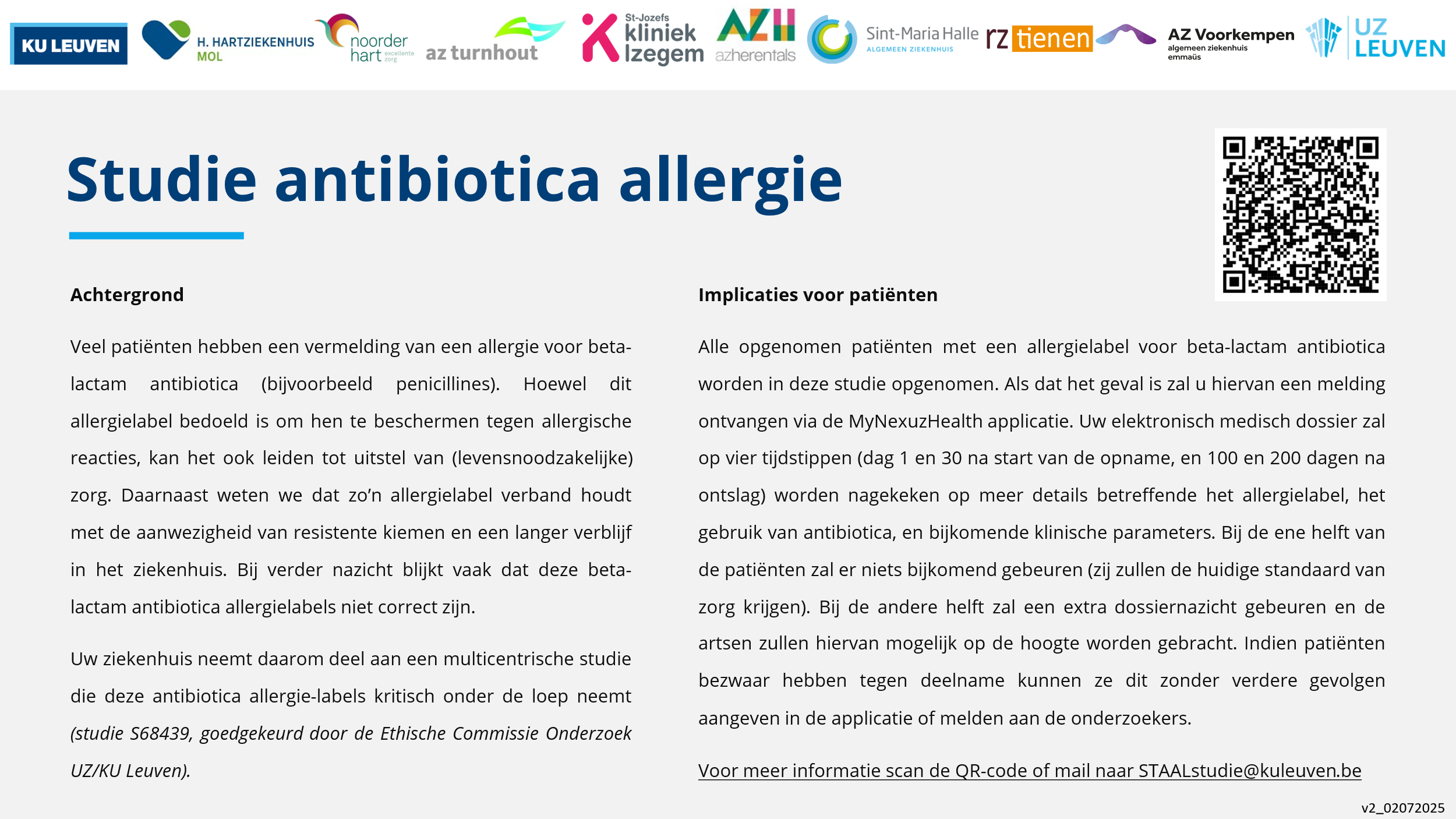


**Figure S2.** Patient notification poster with link to website and information letter. Original Dutch-language patient notification materials used in participating hospitals. These materials inform patients about the quality improvement study and opt-out procedure.

**Figure S3.** Exemplary algorithm for antibiotica allergy label delabeling/refinement.

**
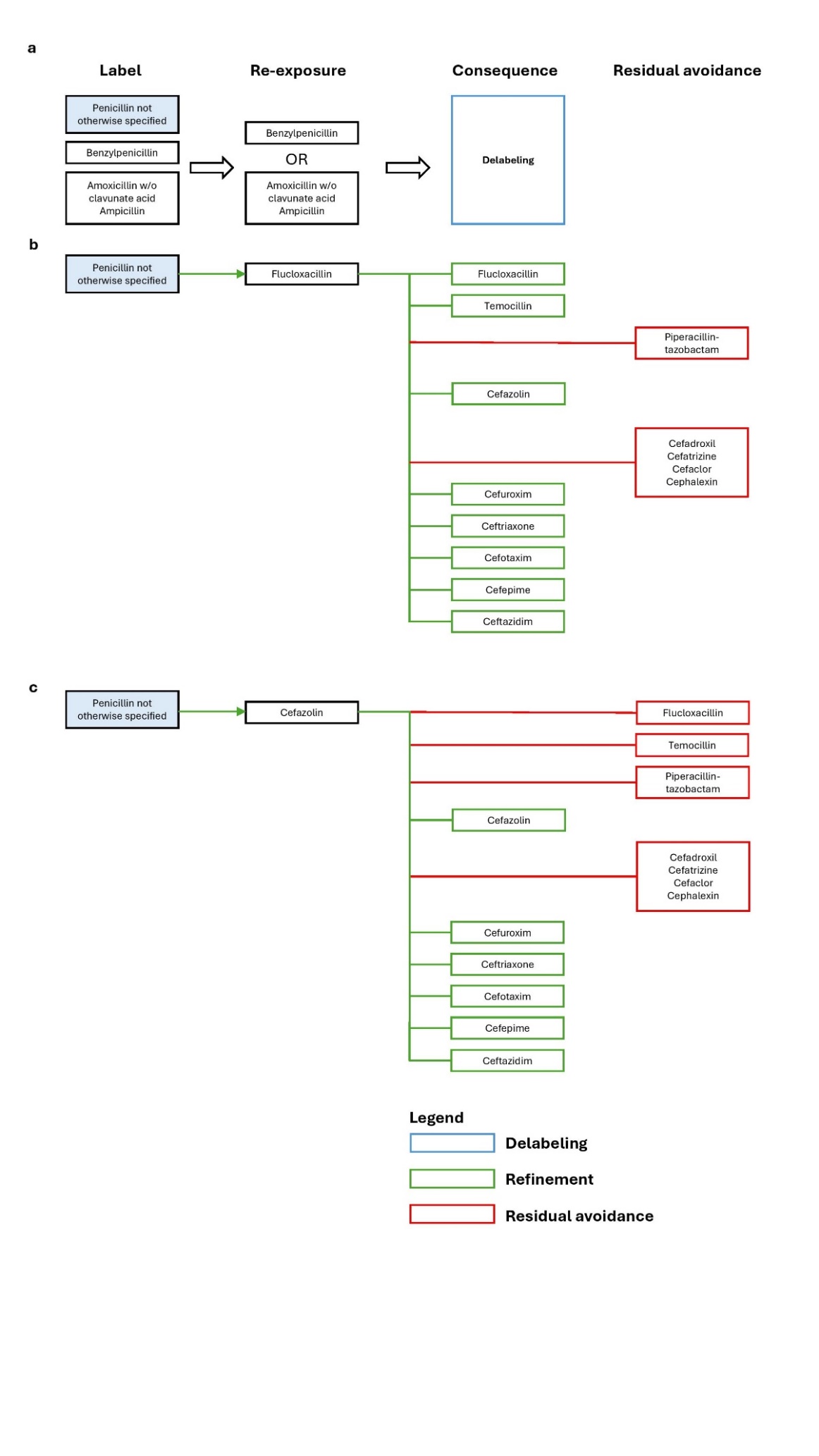
**

**Figure S3**. Algorithm for antibiotica allergy label delabeling/refinement. The initial BLAL on the left, followed by the potential re-exposure (second column) and the consequence (third column) in terms of delabeling and/or refinement (green lines). Antibiotics that remain to be avoided are shown in the fourth column (red lines). In the illustrated examples, aminocephalosporins remain to be avoided.

**Figure S4**. AFC results communication templates (Dutch original).


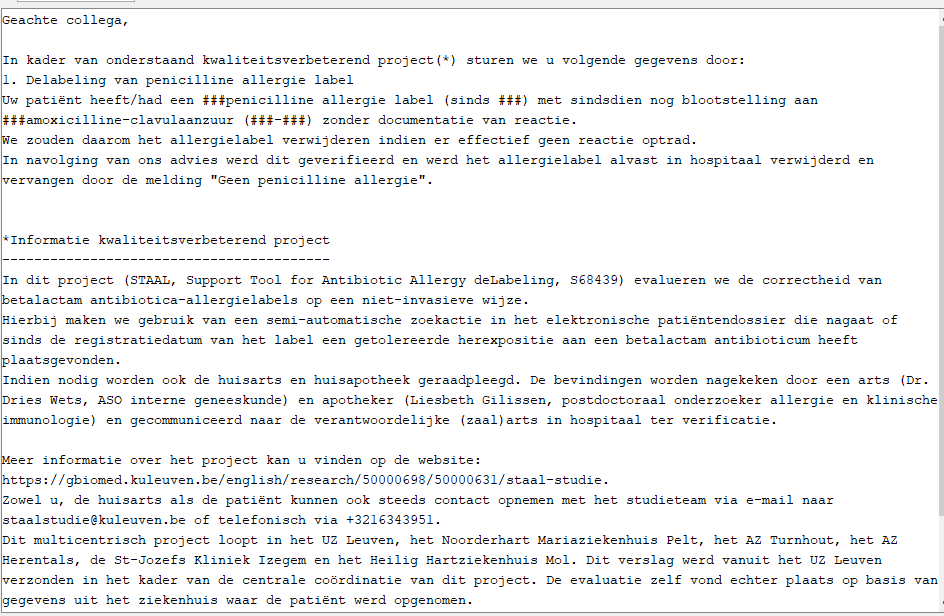


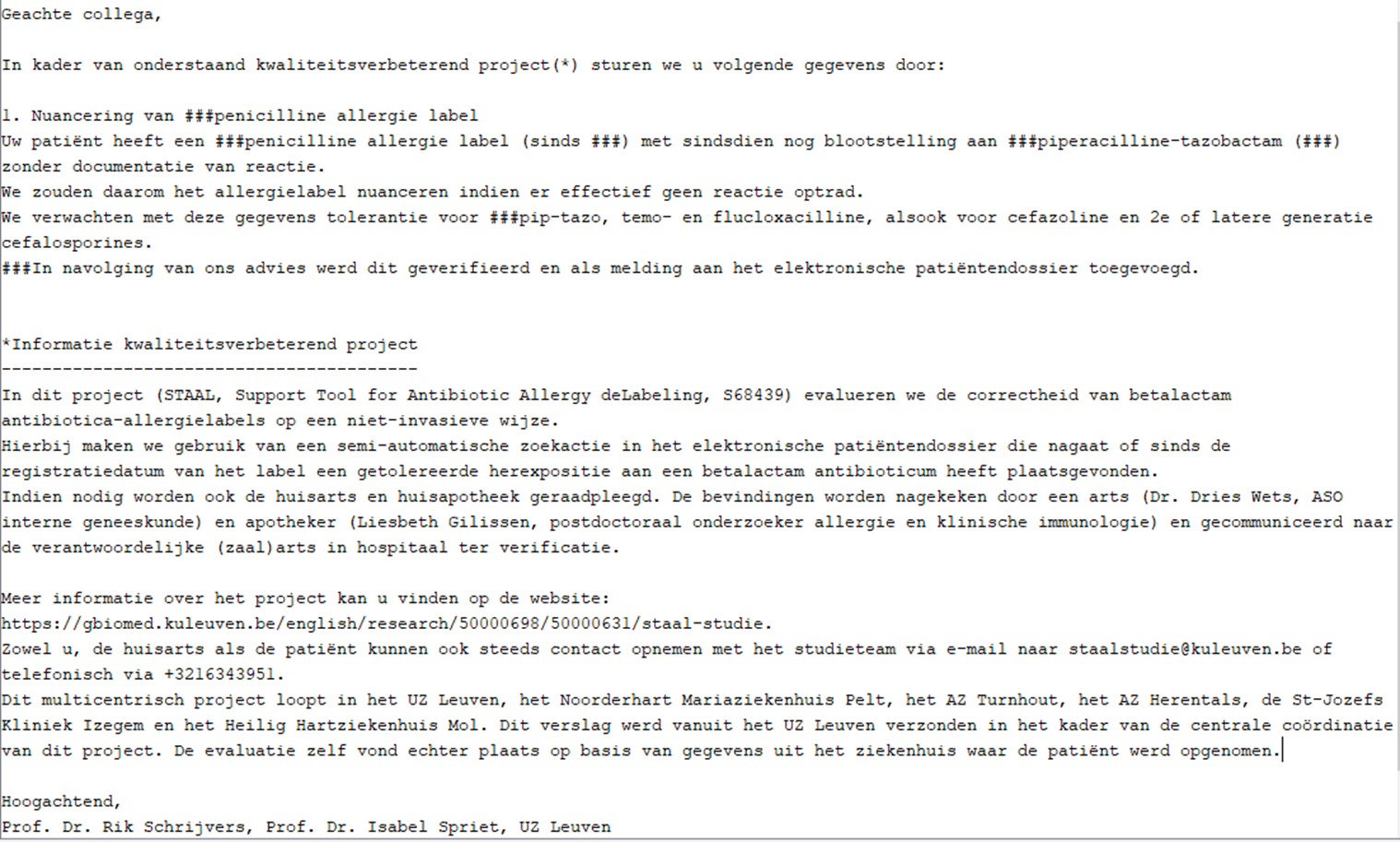


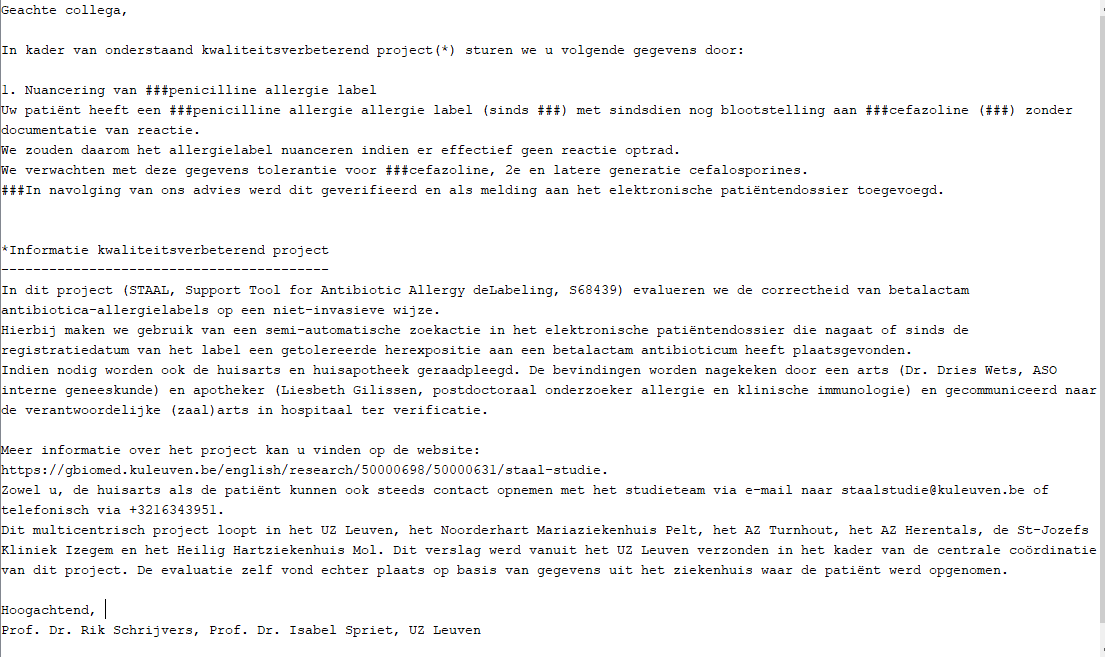


**Figure S4.** Original Dutch-language templates used to communicate AFC verification results and potential allergy label refinement to clinicians and patients. In case of an incorrect BLAL, a study team member records a formal AFC report in the EPR. This report is available for hospital physicians and, after validation by the coordinating investigator, also to the patient via the Mynezuxhealth application. The report is additionally transmitted to the primary care health care provider via secure e-letter through the national eHealth platform. It includes a summary of the findings and, where applicable, a proposed label refinement or removal. Patients are referred to their primary care HCP, the participant information letter and the study webpage for further questions or in case of disagreement with the label adjustment.

**Table S1**. Study flowchart.

| **Procedures/ Assessment** | **Inclusion** | **BLAL fact- check: search for re-exposure** | ***In case of incorrect BLAL :***  **BLAL adjustment** | ***In case of incorrect BLAL :***  **Communication of BLAL fact-check result** | **Follow-up** | **Long-term**  **follow-up** |
| --- | --- | --- | --- | --- | --- | --- |
| Timing (days) | Day 1 | Day 1 | Day 2 | Day 3-7 | Day 30 | Day of discharge + 100, + 200 days |
| Eligible patients are identified by stored query | X |  |  |  |  |  |
| Study member verifies inclusion and exclusion criteria | X |  |  |  |  |  |
| Study member extracts demographics, baseline measurements and details of the BLAL from EPR | X |  |  |  |  |  |
| Study member runs the BLAL-fact-check tool for automated search for re-exposure |  | X |  |  |  |  |
| Study member verifies output of the BLAL fact-check tool |  | X |  |  |  |  |
| Study member contacts primary care physician/pharmacist |  | X |  |  |  |  |
| Study member sends advice to remove or adjust the BLAL to treating physician via ‘follow-up note’ in the EPR |  | (X) |  |  |  |  |
| Study member checks if BLAL is removed/adjusted, and, if not, calls treating physician |  |  | (X) |  |  |  |
| Study member makes formal report of the BLAL fact-check result in the EPR (for hospital physicians and patient) |  |  |  | (X) |  |  |
| Study member sends report to primary care HCP via e-letter |  |  |  | (X) |  |  |
| Study member extracts follow-up data from the EPR |  |  |  |  | X | X |
| Study member performs (Serious) Adverse event (S)(AE) assessment | X | X | X | X | X | X |

Allocation will be performed on hospital level (participating sites) before the start of the study.
The part highlighted in grey only applies for hospitals/patients in intervention phase.
